# Evaluating Effectiveness of mHealth Apps for Older Adults With Diabetes: Meta-Analysis of Randomized Controlled Trials

**DOI:** 10.2196/65855

**Published:** 2025-06-17

**Authors:** Renato Ferreira Leitao Azevedo, Michael Varzino, Erika Steinman, Wendy A Rogers

**Affiliations:** 1 Institute of Gerontology College of Public Health University of Georgia Athens, GA United States; 2 College of Applied Health Sciences University of Illinois Urbana-Champaign Champaign, IL United States

**Keywords:** mHealth apps, diabetes, older adults, RCT, meta-analysis, gerontechnology, geriatric, randomized controlled trial, effectiveness, intervention, blood glucose, management, glycemic control, mobile phone

## Abstract

**Background:**

The global population is aging rapidly, with projections indicating a doubling of older adults by 2050. Among the chronic conditions affecting this demographic, diabetes stands out due to its prevalence and impact on health. Mobile health (mHealth) app interventions show promise in improving health outcomes, leveraging the widespread adoption of smartphones among older adults.

**Objective:**

This meta-analysis aimed to evaluate the effectiveness of mHealth apps specifically tested for older adults with type 1 or type 2 diabetes. It addresses the gap in existing literature by focusing on this age group, aiming to provide insights into the benefits and challenges of these technologies.

**Methods:**

A meta-analysis of randomized controlled trials (RCTs) was conducted across major databases using PRISMA (Preferred Reporting Items for Systematic Reviews and Meta-Analysis) guidelines to examine the effectiveness of mHealth apps for improving older adults’ diabetes outcomes. Primary outcomes included changes in glycated hemoglobin (HbA_1c_), fasting blood glucose, and medication adherence levels. We retrieved 4247 papers, of which 257 were moved to full review, and 7 were identified following our criteria. Papers were excluded if the study was not an RCT, did not examine the effect of mHealth apps, or was not conducted with older adults. We provide a mixed methods perspective, pairing the effect sizes in the literature with a review of features included in these apps, allowing for a more comprehensive comparison and reference for future RCT interventions with similar technologies designed for older adults.

**Results:**

Overall, our results indicated that mHealth app interventions can be effective for managing blood glucose in older adult populations. The 7 RCTs that met the inclusion criteria involved a total of 490 participants. The meta-analysis revealed a significant reduction in HbA_1c_ levels (Hedges g –0.40, 95% CI –0.75 to –0.06) among older adults using mHealth apps. Limited data on fasting blood glucose and medication adherence showed positive trends, echoing the main HbA_1c_ findings.

**Conclusions:**

mHealth apps demonstrated effectiveness in improving glycemic control among older adults with diabetes, highlighting their potential as tools for health management in this demographic. Our effect sizes were comparable with other meta-analyses conducted across different aging groups, suggesting that diabetes mHealth apps can be as effective for older adults compared to younger cohorts. Some data suggests that the effectiveness of mHealth apps might decrease over trial time. These findings underscore the need for further research to refine these interventions and optimize their impact on older adults’ health outcomes. We evaluated each RCT intervention and identified app components that could relate to the app’s effectiveness to guide the design of future diabetes management RCTs and mHealth tools for older adults.

**Trial Registration:**

Open Science Framework 10.17605/OSF.IO/AWVCX; https://osf.io/awvcx

## Introduction

### Background

As of 2024, there are 8.1 billion people living on the planet. The good news is that people worldwide are living longer, and life expectancy has roughly tripled throughout human history [1]. Improvements in hygiene and sanitation, immunization programs, and other health care and medical advancements, as well as healthier lifestyles, have contributed to human longevity. Presently, most people are expected to live into their sixties and beyond. The World Health Organization estimates that every country in the world is experiencing growth in both the proportion of older adults and the population size. By 2030, a total of 1 in 6 people are projected to be aged 60 years or older, and by 2050, the global population of older adults is expected to double (2.1 billion) [2].

However, with increased longevity, the prevalence of chronic conditions commonly linked with advancing age has become a significant public health concern. For example, diabetes is increasingly becoming a prevalent health concern among older adults. In 2019, global estimates indicated that 19.3% of people aged 65-99 years (135.6 million) live with diabetes [3]. This number is projected to reach 195.2 million by 2030 and double by 2045; approximately 276.2 million older adults with diabetes [3-5]. In the United States alone, 38.4 million people have diabetes, and 97.6 million have prediabetes (11.6% and 38% of the country’s population, respectively) [4,6]. Furthermore, diabetes is 1 of the top 5 most common chronic conditions impacting older adults (≈27% of adults aged 65+ years) [7]. Hence, among various chronic conditions affecting older adults, diabetes stands out as one of the most pervasive, highly prevalent comorbidities among patients and significantly compromising the health and wellness of a large segment of the aging population.

### Potential for Mobile Health Interventions

Most forms of diabetes are chronic, but all forms are manageable with medications and lifestyle changes [8,9]. Moreover, diabetes is considered one of the major controllable risk factors for cardiac, brain, eye, and kidney diseases [9,10]. With the continuous advances of technology, the use of mobile health (mHealth) tools to facilitate the attainment of health objectives (ie, continuous glucose monitoring and medication adherence) is increasing and holds the potential to revolutionize health care delivery worldwide [11-13], see also the study by Wilson et al [14]. This transformative shift is leveraged and propelled by advancements and wide availability in mobile technologies and applications, the emergence of opportunities for integrating mHealth into existing eHealth services, and the ongoing expansion of coverage in mobile cellular networks.

Advancing technologies are becoming increasingly more affordable, and smartphone ownership among older adults is rising from only 10% in 2011 to 76% in 2023 [15]. Furthermore, relying on smartphones for internet access is notably prevalent among older adult Americans with lower household incomes and individuals with less formal education. Surveys conducted from 2013 to 2023 demonstrated an increased smartphone dependency among older adults, with numbers rising from 3% in 2013 to 16% of users aged older than 65 years depending on their phones for internet access [15]. Therefore, because health information is more often only accessible electronically, such as through electronic health records on patient portals, it is reasonable to conclude that a significant portion of older adults will use their smartphones for access to health information. Moreover, a systematic review conducted by Wilson et al [14] indicated that smartphones and tablets were seen by older adults with and without cognitive impairment as acceptable, enjoyable, and valuable alternatives to conventional assistive technology.

As per the data accessible up to mid-2024, an estimated 35,019 to 41,430 health care and medical apps are available on the Apple App Store [16], and between 36,260 to 57,209 apps are available on the Google Play Store [17]. Although mobile apps continue to expand in different markets (eg, social, communication, health, entertainment, banking, and personal finance), they are not always designed for every age demographic and there is a need to investigate their effectiveness, as well as to advance guidelines for designing these apps specifically for older adults [11,18-20].

mHealth apps can be particularly relevant for older adults with diabetes due to several reasons, including: continuous blood glucose (BG) monitoring and data tracking, personalized medication and insulin reminders, enhanced communication with health care providers, education and support (ie, guidance on nutrition, exercise, and lifestyle changes), emergency alerts and risk prevention (ie, hypoglycemia and hyperglycemia alerts), as well as by providing tools that empower older adults to take a more active role in their own health, promoting independence and improving quality of life.

### Existing Research and Why Are Meta-Analyses Important?

Meta-analyses are vital for evidence-based practice and health care decision-making because they use an objective qualitative approach for assembling, arranging, and assessing existing literature in a research domain, which also includes a quantitative approach and statistical analysis of a collection of results from its individual studies for the purpose of integrating findings [21-23]. The process involves thorough searches to identify relevant articles for statistical analysis, aiming to minimize bias and ensure a true representation of available research.

The development of mHealth apps involves a substantial undertaking, requiring a significant allocation of resources, which is also true for researchers conducting randomized controlled trials (RCTs). The development of mHealth apps encompasses not only financial investments but also considerable amounts of time, technical expertise, and interdisciplinary collaborations to ensure that these applications are user-friendly and effective. The complexity of rigorous testing and validation contribute to the resource-intensive nature of mHealth application development. However, the potential benefits concerning improving health care access, patient engagement, and the overall quality of health care services make this investment in resources valuable and necessary for the future of health care technology.

Hence, conducting a comprehensive meta-analysis is crucial to provide information for cost-benefit analysis and to identify health technology components and features that are effective for the intended users. Given the uncertainty surrounding the efficacy of these applications, particularly for older adults, a gap in consolidating the existing knowledge to propel the field forward is justified. To our knowledge, there has not been a meta-analysis to evaluate the effectiveness of mHealth apps for improving older diabetic adults’ health outcomes. The identified meta-analyses evaluated mHealth apps on a wider age spectrum and do not evaluate the effectiveness for older adults [10,24-29], but see the study by Robert et al [30] for eHealth nutrition intervention. The studies by Mao et al [10] and Iribarren et al [26] assessed the effectiveness of mHealth apps for managing both diabetes and hypertension, finding that these apps significantly reduced glycated hemoglobin (HbA_1c_) [10,27] and fasting blood glucose levels [10]. Eberle et al [24] conducted a scoping review and reported improvements in HbA_1c_ levels for individuals with type 1 and those with type 2 diabetes, along with positive trends in self-care behaviors. Focusing on educational mHealth interventions, Nkhoma et al [28] support these findings, showing the benefits of these technologies for patients with type 1 and those with type 2 diabetes, with improvements in HbA_1c_ levels. Hyun et al [25] compared the effectiveness of mHealth apps with and without e-coaching support from health care providers, as well as standard diabetes care. Their findings indicated that while the combination of an app and e-coaching was more effective at 3 months, the app alone led to greater reductions in HbA_1c_ levels at 6 months. Tchero et al [29] examined the effectiveness of telemedicine compared to standard care, incorporating some support for mHealth apps’ effectiveness by finding reductions in HbA_1c_ scores. Additionally, Iribarren et al [26] explored mHealth apps more broadly for health promotion and disease management, including studies relevant to diabetes. The authors identified key app features that were most beneficial, such as reminders, in-app communication, gamification elements, journaling capabilities, and goal-setting functions.

While these meta-analyses [10,24-29] provide a thorough overview of the availability and effectiveness of mHealth apps for diabetes management and the identification of possible app features contributing to these benefits [26], they do not specifically address the unique needs of older adults. Hence, the evidence concerning the effectiveness of mHealth apps for older adults with diabetes is scarce and fragmented and often extrapolated from studies of younger age groups.

### Significance

Our goal was to address the pressing need to evaluate the effectiveness of mHealth apps tailored specifically for older adults with diabetes. The overarching purpose was rooted in the understanding that diabetes not only affects a substantial number of older adults but is intricately linked to other health complications, thereby compromising their overall quality of life. We addressed this research gap by concentrating on the studies conducted directly with older adults, with the ultimate goal of shedding light on the potential benefits and challenges associated with mHealth apps and to evaluate the effectiveness of these promising health technologies on improving older adults’ health management and outcomes. Older adults often struggle with multiple chronic conditions, each demanding careful attention to medication adherence, lifestyle adjustments, and continuous monitoring, and often have distinct needs, facilitators, and barriers to using health technologies, such as mHealth apps.

Furthermore, conducting meta-analyses to determine effect sizes in the existing literature—even in the earlier formative stages of accumulated knowledge, when only a limited number of interventions have been published—is crucial for estimating statistical power in the context of future health technology interventions, because it provides a consolidated understanding of the cumulative evidence at this stage and enables researchers to make informed decisions about the feasibility and potential impact of such interventions in this population. It offers the opportunity for researchers to adapt their ongoing projects based on the anticipated effect sizes and statistical power, allowing them to proactively allocate resources and address potential challenges (eg, alteration of funding support and recruitment challenges) by determining minimum sample sizes necessary for the planned analyses, ultimately ensuring the success and relevance of these critical health technology projects.

We aimed to inform guidelines and practices by harnessing the capabilities of smartphones and wearable devices. These applications offer real-time monitoring, personalized feedback, and educational resources, facilitating better self-management practices among older individuals with diabetes. Our analyses provided insights for the design and implementation of technology to enhance health management and well-being for older adults. It complements other systematic reviews that focused on psychological techniques implemented in mHealth solutions [31] and design guidelines of mobile apps for older adults [11], providing a perspective of features available in these mHealth app interventions. Thus, we provided a mixed methods perspective, pairing the effect sizes in the literature with a review of features included in these apps, allowing for a more comprehensive comparison and reference for future RCT interventions with similar technologies designed for older adults.

### Research Objective

The primary objective of this systematic review and meta-analysis was to analyze the literature to determine the effect of mHealth apps on diabetes (type 1 or type 2) management for older adults. This analysis drew upon RCT interventions conducted directly with older adults who adopted a mHealth app to improve outcome measures related to diabetes to evaluate its effectiveness.

### Research Question

Do mHealth apps improve older adults’ diabetes management?

## Methods

### Design, Data Sources, and Literature Search Strategy

In December 2022, we conducted a systematic literature search in the Clinical Trials Cochrane Library, EBSCO, ProQuest, PubMed/MEDLINE, ScienceDirect, Scopus, and Web of Science per the PRISMA (Preferred Reporting Items for Systematic Reviews and Meta-Analyses; checklist provided in Multimedia Appendix 1) strategy [32,33]. These databases are representative of the health-related literature and are the largest databases in the field. The search strategy included keywords that combine (1) mHealth apps terms (eg, app, cell phone, digital technolog*, etc), (2) age-related terms (eg, aging, elderly, geriatric*, older adults, etc), and (3) health conditions related terms (eg, diabet*, gluco*, glycemic*, A_1C_, etc). We developed our search strategies in consultation with a search specialist and developed keywords in consultation with a subject matter expert in pharmacy and geriatrics. This list of keywords was cross-validated, as a quality control, against the column “keywords” on the Cochrane Library database to ensure that we were not missing relevant keyword variations. For our general search strategy query strings and complete list of keywords, see Multimedia Appendix 2. Additionally, we manually searched reference lists and previous meta-analyses to identify further papers. We used ascendancy and descendancy approaches on the full papers selected as quality control, using the sources cited in our included sources and the Crossref (Crossref Org) and CoCites [34] systems. This study protocol was registered on OSF Registries.

### Inclusion and Exclusion Criteria

Studies were included in the review if they (1) were RCTs, (2) examined the effects of mHealth app-based self-care interventions relative to other control conditions (eg, usual care) on patient outcomes, (3) included people with type 1 or type 2 diabetes, (4) included older adults (60 years or older), and (5) were published in English-language peer-reviewed journals. We excluded review articles, case reports, book chapters, and studies that only provided an abstract. We further excluded studies that only reported protocols without data or only outcomes on app use (ie, app satisfaction and use rate), as the goal of this meta-analysis was to evaluate direct outcomes related to the effectiveness in improving BG control. Based on the SPIDER (Sample, Phenomenon of Interest, Design, and Research) framework for qualitative evidence synthesis [35], we outline our inclusion criteria as follows: (1) sample: older adults (60 or older) with type 1 or type 2 diabetes, (2) phenomenon of interest: the effect of mHealth app interventions on BG control, (3) design: RCTs, (4) evaluation: effectiveness in improving BG control, and (5) research type: mixed methods (eg, quantitative: effect sizes of outcome measures; qualitative: features of the mHealth apps that are effective to the target population).

### Study Screening and Selection

We carried out the process of importing and compiling citations using Microsoft Excel (Microsoft Corp) for initial selection and screening. Three researchers (RFLA, MV, and ES) were involved in the screening and selection of studies. RFLA removed duplicate entries and provided training to both MV and ES. In the initial phase, MV and ES independently assessed the relevance of study titles. Following the title screening, MV and ES screened the abstracts to identify potentially relevant studies. Any disagreements (71/1341, 5.23%) that arose during these stages were resolved through consensus supervised by RFLA. Subsequently, RFLA and MV retrieved and assessed full texts of potentially relevant papers against the inclusion and exclusion criteria. Any areas of disagreement (58/257, 22.6% of records) were collectively compared and discussed. Additionally, the snowballing method was used by examining the reference lists of relevant articles and other meta-analyses involving mHealth apps with a broader age range.

Throughout the screening process, we relied solely on information from the title and abstract fields for classification. Author names, publication year, and journal information were available for reference if needed to retrieve missing abstracts or confirm duplicates for removal but were not actively used for screening purposes to mitigate biases.

### Outcome Measures

The following outcome measures are most usually reported in the diabetes literature: (1) HbA_1c_, also referred to as A_1c_, (2) fasting blood glucose (FBG), also referred to as fasting blood sugar (FBS), and (3) medication adherence.

When available, we reported other secondary outcome measures, such as measures of diabetes knowledge, to provide a comprehensive perspective of the impact and efficacy of mHealth apps in the management of diabetes for older adults.

### Data Extraction, Synthesis, and Analysis

We extracted the following information about each study: author, publication year, study location, intervention and control groups, baseline and follow-up outcome variable values (ie, HbA_1c_, FBG, and medication adherence), sample size, mean age of participants, sex ratio, trial duration length, mHealth app features, and main findings related to the outcomes of interest.

We synthesized the studies based on their outcomes, as the clinical approach prioritizes enhancing outcomes for older adults through mHealth RCT interventions. We conducted a meta-analysis to assess the impact of the interventions on the participants’ BG levels and diabetes medication adherence. To assess changes in BG levels and medication adherence, we combined the identified studies featuring intervention groups (using mHealth apps) and control groups (receiving usual care). We computed the mean differences, accompanied by a 95% CI and effect sizes (Hedges *g*) in R (version 4.2.1; R Foundation), with the packages *meta* [36] and *esc* [37]. Data comprised studies reporting modifications in HbA_1c_, FBG, and medication adherence, comparing baseline values to the conclusion of the study for both intervention and control groups. HbA_1c_ is the most important and most studied clinical outcome related to diabetes management [24]. Plots were created with the packages *meta* [36] and *robvis* [38,39].

### Critical Appraisal (Assessment of Quality of Evidence and Risk of Biases)

**Figure 1:**
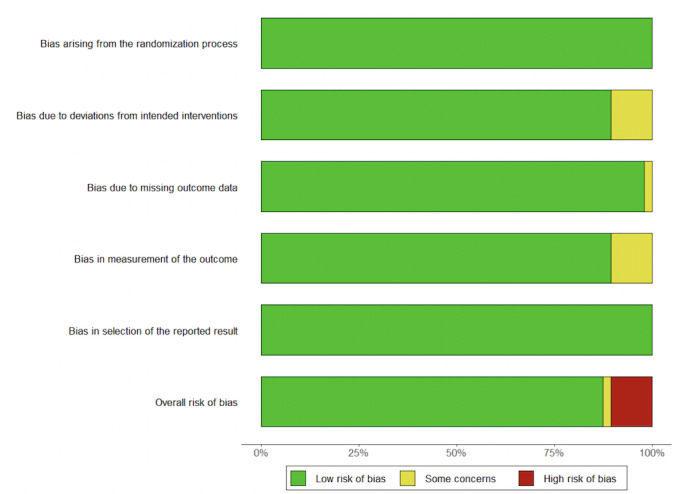
Bias appraisal across domains.

Based on the Cochrane Risk of Bias 2.0 tool for randomized trials [40], 2 researchers (RFLA and MV) evaluated the risks of biases in included RCTs across 5 specific dimensions: D1: randomization process, D2: deviations from intended interventions, D3: missing outcome data, D4: outcome measurement, and D5: selection of reported results. Risk ratings of “low,” “some concerns,” and “high” were assigned to each bias (see also Guyatt et al [41] and Figure 1 for a global risk assessment across all identified studies). RFLA and MV had an interrater agreement of 97.14%. After reviewing the single disagreement case involving 1 category in 1 of the papers, both authors reached a consensus to adjust the rating from low concern to some concerns. In Multimedia Appendix 3, we provide additional information concerning publication bias and sensitivity analysis.

### App Feature Analysis

To offer a comprehensive analysis of the identified mHealth apps, we supplemented the quantitative assessment, as demonstrated by the calculation of effect sizes, with a qualitative exploration of the most prevalent components and features comprising these app interventions. Our mixed methods approach aimed to contribute to future design and recommendations by identifying crucial components and comparing the effect size findings with an understanding of which features may explain the success of these interventions. Conversely, the absence of a feature could account for the lack of success observed in certain apps and RCT interventions. The categories for this analysis were formulated by adapting the conclusions drawn from the research conducted in other studies [26,42-44]. Table 1 presents a summary of all features identified across the 7 papers and respective studies.

**Table 1:** Mobile health (mHealth) app features.

Study			LOG-TRA^a^		MON-VIS^b^		REM^c^		EDUC^d^		PERSO-GOAL^e^		REW-GAME^f^		SOCIAL^g^		CONNECT^h^		GLUCOM^i^
Takenga et al [45]			Yes		Yes		UNK^j^		UNK		Yes		UNK		UNK		Yes		Yes
Or and Tao [46]			Yes		Yes		Yes		Yes		UNK		UNK		UNK		UNK		Yes
**Dugas et al [42]**																			
	Int^k^ 1 (app only)	Yes		Yes		UNK		Yes		No		Yes		No		No		No	
	Int 2 (app + provider)	Yes		Yes		UNK		Yes		No		Yes		No		Yes		No	
	Int 3 (app + social team)	Yes		Yes		UNK		Yes		No		Yes		Yes		No		No	
	Int 4 (app + provider + social team)	Yes		Yes		UNK		Yes		No		Yes		Yes		Yes		No	
Sutema et al [47]			Yes		No		Yes		No		No		No		No		No		UNK
Sun et al [48]			Yes		UNK		Yes		UNK		UNK		UNK		No		Yes		Yes
Esferjani et al [49]			No		UNK		No		Yes		UNK		UNK		Yes		Yes		UNK
Poonprapai et al [50]			Yes		Yes		Yes		Yes		UNK		Yes		Yes		Yes		UNK

^a^LOG-TRA: logging and tracking.

^b^MON-VIS: monitoring and visualizations.

^c^REM: reminders.

^d^EDUC: education.

^e^PERSO-GOAL: personalized goal setting.

^f^REW-GAME: reward system and gamification.

^g^SOCIAL: social support.

^h^CONNECT: connection to health practitioners.

^i^GLUCOM: integration with glucometers.

^j^UNK: unknown.

^k^Int: intervention

## Results

### Study Selection

**Figure 2:**
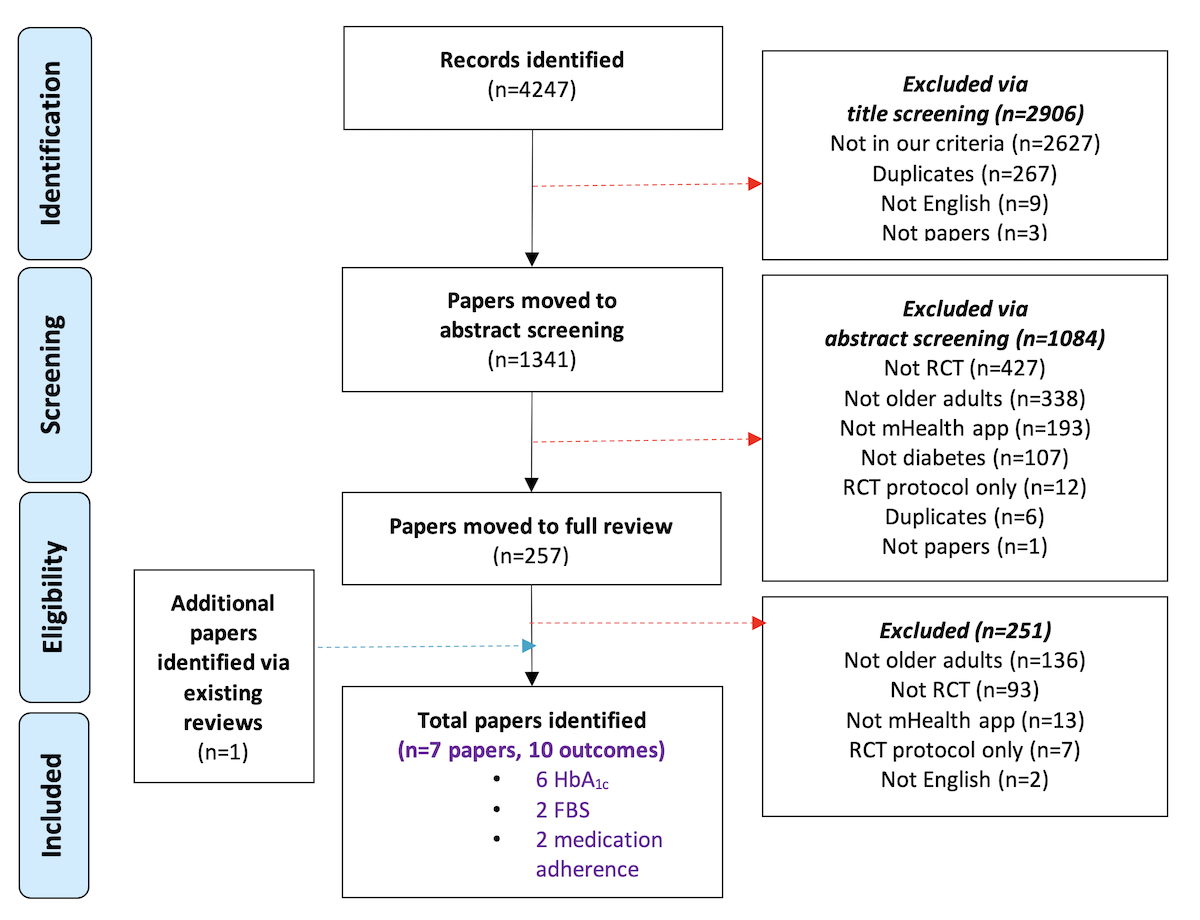
PRISMA flowchart of studies included in the review. FBS: fasting blood sugar; HbA1c: glycated hemoglobin; PRISMA: Preferred Reporting Items for Systematic Reviews and Meta-Analysis; RCT: randomized controlled trial.

**Figure 3:**
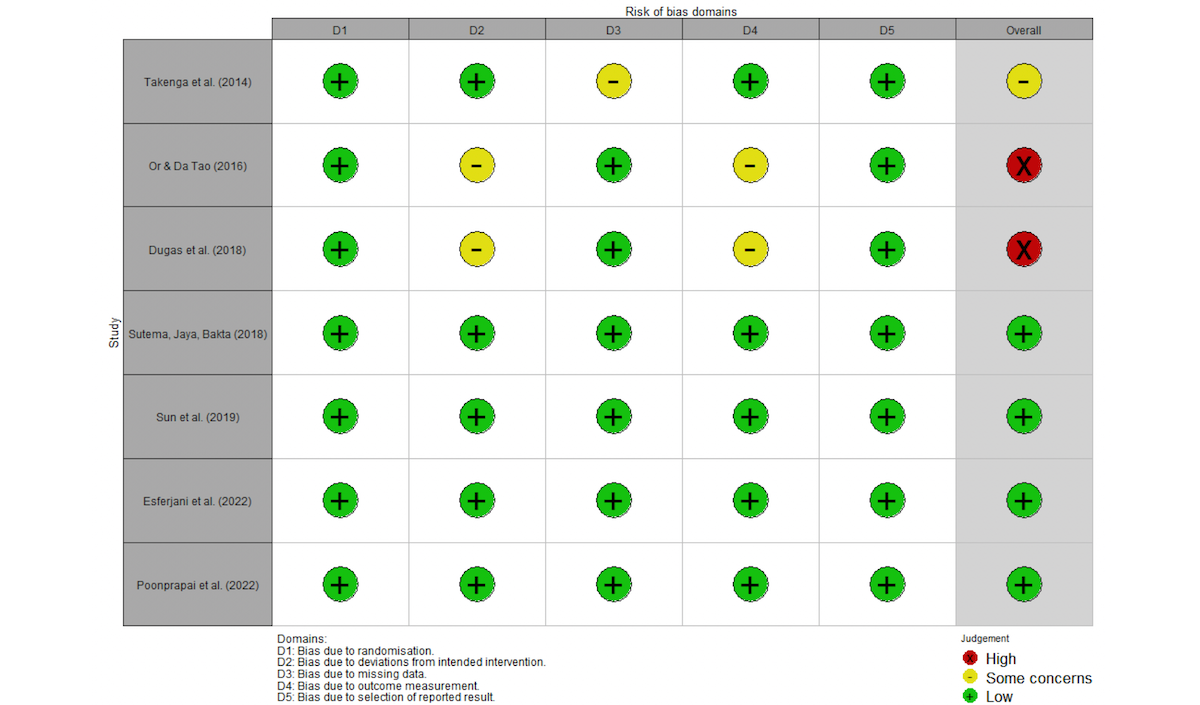
Risk of bias assessment summary (version 2.0) [42,45-50].

We retrieved 4247 papers, and 2 reviewers (MV and ES) completed a full screen review of 257. We excluded papers if the study was not an RCT, did not examine the effect of mHealth apps, or was not conducted with older adults, resulting in 7 papers included in the final analysis (Figure 2). The meta-analysis incorporated a total of 490 participants from these 7 papers. The characteristics of the included studies are presented in Table 2. These studies were undertaken in China, Hong Kong, Indonesia, Iran, the Republic of Congo, Thailand, and the United States and were published from 2014 to 2022. During this period, there was an upward trend in sample sizes (Table 2), and studies enhanced their methodologies, as indicated by our Cochrane assessment (Figure 3 [42,45-50]).

**Table 2:** Study characteristics and demographics.

Study ID	Sample (n, age)	Country	Diabetes eligibility	Study length	Data collection points	Group description	Outcome
Takenga et al [45]	Control: n=5 (5 male), mean age 65.8 (SD 5.97, range 60-75) y Intervention: n=5 (4 male), mean age 62.8 (SD 4.2, range 60-70) y	Democratic Republic of Congo	Type 2 diabetes diagnosis	60 days	Baseline and 60 days	Conventional therapy without the use of telemedicine Treatment with the use of the telemedicine “Mobil Diab” app	HbA_1c_^a^, blood glucose
Or and Tao [46]	Control: n=11, mean age 69.7 (SD 10.2) y Intervention: n=14, mean age 69.3 (SD 9.7) y	Hong Kong	Diabetes diagnosis	3 months	Baseline and 3 months	A logbook for manually recording readings and a 2-in-1 blood glucose and blood pressure monitor device Digital tablet computer-based system with a 2-in-1 blood glucose and blood pressure monitor device	HbA_1c_, FBG^b^, diabetes knowledge
Dugas et al [42]	Control: n=5, mean age 66.40 (SD 4.93) y Intervention 1: n=5, mean age 65.40 (SD 4.72) y Intervention 2: n=5, mean age 72 (SD 9.3) y Intervention 3: n=6, mean age 66 (SD 5.18) y Intervention 4: n=6, mean age 68.17 (SD 3.66) y	United States	Type 2 diabetes>3-year diagnosis and HbA_1c_ >7.9	13 weeks	Baseline and 90 days	Usual care Using the app without clinician or peer engagement Using the app with clinician engagement features Using the app with peer engagement features Using the app with both clinician engagement and peer engagement features	HbA_1c_
Sutema et al [47]	Control: n=31 (17 male), mean age 63.48 (SD 3.57) y Intervention: n=31 (20 male), mean age 62.97 (SD 4.94) y	Indonesia	Diabetes diagnosis, undergoing diabetic neuropathy therapy	4 weeks	4 weeks	Neuropathic pain therapy usual care Neuropathic pain therapy, usual care, and medicine reminder app	Medication adherence
Sun et al [48]	Control: n=47 (18 male), mean age 67.9 (66-71) y Intervention: n=44 (19 male), mean age 68.04 (66-72) y	China	Diabetes diagnosis, HbA_1c_ >7% and <10%	6 months	Baseline, 3 months, and 6 months	Dietary guidance from dietitians in person at baseline and after the study procedures A mHealth management app and glucometer to aid data transmission from the user to the dietitian. Once-monthly dietary recommendations based on app information.	HbA_1c_, FBG, and PBG^c^
Esferjani et al [49]	Control: n=59, age 60 to 64 (67.8%) y and 65 to 70 (32.2%) y Intervention: n=59, age 60 to 64 (62.7%) y and 65 to 70 (37.3%) y	Iran	Diabetes diagnosis	3 months	Baseline and 3 months	Routine care from the health center (an approximately 20-minute visit every 3 months) with a doctor, a dietitian, and a nurse Three sessions of educational content delivered via a WhatsApp group app on mobile devices and a printed booklet containing health information	HbA_1c_
Poonprapai et al [50]	Control: n=79 (40.5% male), mean age 67.80 (SD 6.18) y Intervention: n=78 (39.7% male), mean age 67.36 (SD 5.72) y	Thailand	Diabetes diagnosis, HbA_1c_ >7% or 53 mmol/mol	9 months	Baseline, 3 months, 6 months, and 9 months	Usual care A mobile app to deliver diabetes care information to family caregivers of a diabetes patient	HbA_1c_, medication adherence, diabetes knowledge

^a^HbA_1c_: glycated hemoglobin.

^b^FBG: fasting blood glucose.

^c^PBG: postprandial blood glucose.

Of these papers, the study by Dugas et al [42] had 4 different app interventions, testing the use of their app without clinician or peer engagement, with only clinician engagement, with only peer engagement, and with both clinician and peer engagement. Note that Takenga et al [45] extended their study to encompass a broader age spectrum. Nonetheless, because the authors provided effects for each participant, we computed the appropriate effect sizes specifically for the older adults within their sample, enabling us to include the study in our meta-analysis.

### About HbA1c

**Figure 4:**
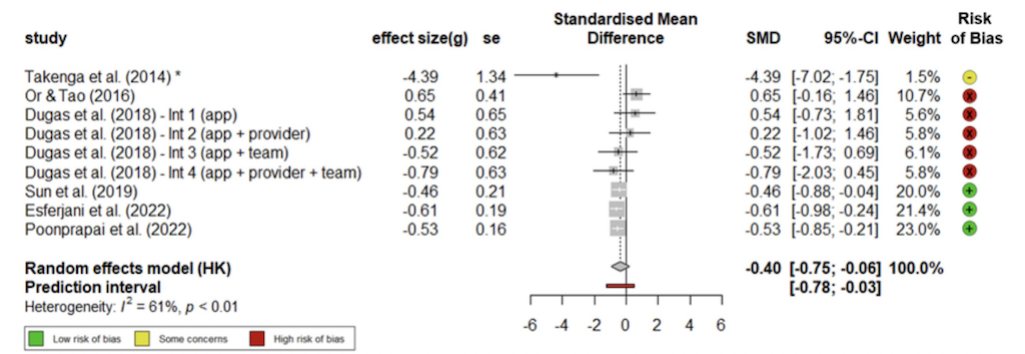
Forest plot of standardized mean differences of mHealth apps on HbA1c reduction [42,45,46,48-50]. HbA1c: glycated hemoglobin; HK: hypertension knowledge; Int: intervention; SMD: standardized mean difference.

The estimated effect sizes indicated that mHealth apps can be effective for older adults managing diabetes, with significant reductions in HbA_1c_ (Hedges *g* –0.40, 95% CI –0.75 to –0.06; see Figure 4 [42,45,46,48-50]). This effect size is comparable with other meta-analyses conducted across different aging groups (HbA_1c_ effect [mean difference: –0.63, 95% CI –0.93 to –0.32, for type 1 diabetes, and –0.54, 95% CI –0.80 to –0.28, for type 2 diabetes [24]); HbA_1c_ effect (g –0.44, 95% CI –0.59 to –0.29 [27]); HbA_1c_ (weighted mean difference: –0.39, 95% CI, –0.50 to –0.29 [10]); HbA_1c_ effect (g –0.48, 95% CI –0.66 to –0.29 [28]), and HbA_1c_ effect (g –0.37, 95% CI –0.43 to –0.31 [29])).

Furthermore, a decrease of –0.4 on the HbA_1c_ scale could be deemed clinically significant, given that 0.5 marks the threshold difference between normal (below 6%) and diabetic ranges (6.5% or above) for diagnosis. For instance, in the prediabetic stage, which falls between 6% to 6.4%, a reduction of 0.4 would transition a patient diagnosis from prediabetic to normal. The variance in the underlying effects across studies was estimated at τ^2^=0.11 (95% CI 0.0505-6.7925), with an *I*^2^ value of 61.1%. This score suggests a moderate to substantial heterogeneity among the study results. We note that this heterogeneity is driven largely by the effects reported in Takenga et al [45] (Multimedia Appendix 3). If we recalculate the *I*^2^ statistic without the study by Takenga et al [45], an estimate of 41.1% will reflect that some variability exists among the included studies, but it was not excessively high.

### About FBG

**Figure 5:**
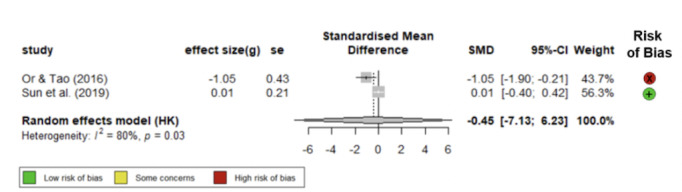
Forest plot of standardized mean differences of mHealth apps on FBS reduction [46,48]. FBS: fasting blood sugar.

Only 2 of the identified studies reported FBG as an outcome measure, namely Or and Tao [46] and Sun et al [48]. Echoing the findings on HbA_1c_, our estimated effect size similarly suggested that mHealth apps may contribute to a significant reduction in FBG levels (g –0.29, 95% CI –1.52 to 0.42; Figure 5 [46,48]). Nevertheless, the scarcity of studies assessing this outcome measure and the wide CI estimate and substantial heterogeneity of these 2 studies (*I*^2^=79.6%) do not provide us with a conclusive picture. Although Or and Tao [46] observed a decline in FBG, Sutema et al [47] did not report significant results for that measure.

### Medication Adherence

**Figure 6:**
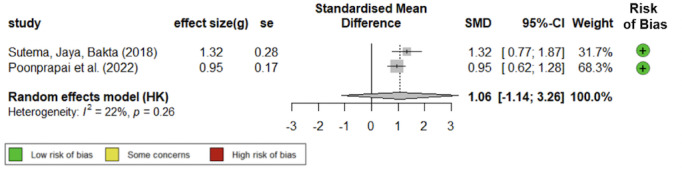
Forest plot of standardized mean differences of mHealth apps on improving medication adherence [47,50].

Medication adherence is a crucial element in managing chronic conditions, but only 2 studies, namely Sutema, Jaya, Bakta [47] and Poonprapai et al [50], assessed that outcome. These RCT studies were well-designed, with a low risk of biases and low heterogeneity (*I*^2^=21.6%), and indicated that mHealth apps could support improvements in medication adherence (Hedges *g* 1.06, 95% CI –1.14 to 3.26; Figure 6 [47,50]). Although Dugas et al [42] presented some medication adherence data, we were unable to calculate an effect size to evaluate the comparative effectiveness of their app because medication adherence data were solely accessible through their app and not available for the control condition.

### Other Diabetes-Related Outcome Measures

Sun et al [48] was the sole study identified that offered measurements for postprandial BG test, which is a measure of glucose in the blood after consuming a meal. They found that after 6 months of their app intervention, participants significantly reduced their postprandial BG levels (Hedges *g* –0.67, 95% CI –1.09 to –0.25) compared to usual care.

Two of the seven papers incorporated assessments of diabetes knowledge [46,50]. Or and Tao [46] used a modified Michigan Diabetes Knowledge Scale [51], comprising 18 true or false statements, scored per percentage correct, to evaluate participants’ knowledge. After 3 months of app usage, no significant differences were observed between intervention and control groups, as diabetes knowledge scores increased for both groups (intervention group, n=14, from 37.3% to 44.1%; control group, n=11, from 43.4% to 51.5%); reflecting increases in diabetes knowledge of 6.8% and 8.1%, respectively. The authors stated that participants in both groups had similar levels of health literacy at baseline. Although the study mentioned an educational component in their app, no data were presented specific to its usage. Alternatively, the control condition might have provided educational information, potentially explaining the absence of differential educational benefits. In contrast, the study by Poonprapai et al [50] assessed participants’ diabetes knowledge using the General Knowledge of Patients with Diabetes [52]. Once again, both intervention and control groups demonstrated increased knowledge over the trial duration, with notable between-group differences emerging after 9 months (mean increase 2.22 pt, 95% CI 1.56 to 2.89, on a 21-item scale, *P*<.001) in favor of the mHealth app intervention, despite similar baseline performances. Though it is not advisable to make definitive conclusions from a single study, this outcome could offer an optimistic reference for designers and researchers that prolonged exposure to educational materials within a mHealth app (ie, lasting 9 months) may lead to increased knowledge on diabetes for older adults beyond the knowledge gains of usual care conditions. For comparison, the study by Nkhoma et al [28], based on 3 studies conducted with a wider age range, found that digital interventions for diabetes improved diabetes knowledge (Hedges *g* 1.003, 95% CI 0.068-1.938).

### Features of mHealth App Interventions

We reviewed each intervention and the app components that may impact effectiveness. This analysis aimed to guide the development of future diabetes management RCTs and inform the design of other mHealth tools tailored for older adults. Out of the 7 papers, 3 (42.9%) explicitly mentioned the integration of their apps with a glucometer device to facilitate the monitoring of BG levels for users. This integration between app and glucometer devices could explain the observed positive effects in reducing glucose levels, as Miller et al [12] showed that the real-time continuous glucose monitoring is indeed effective in reducing glucose levels among older adults, which is an encouraging result given that Miller et al [12] found sustained benefits over 12 months of monitoring use.

Only Takenga et al [45] mentioned the use of personalized goal settings as a behavioral strategy adopted and a feature implemented in their app. Except for Esferjani et al [49], where their app served solely as a platform for delivering educational information and videos, rather than for inputting data, all other interventions (n=6, 85.71%) incorporated features enabling research participants to log and monitor their usage within the app. This prevalent inclusion is expected, as such basic functionality is commonplace across various apps. Four of 7 (57.14%) studies included data visualizations and monitoring components, enabling participants to receive feedback based on the input data. Additionally, 4 of the 7 (57.14%) studies explicitly referenced having a reminder feature (ie, alarms or notifications), and the same proportion (4/7, 57.14%) was found for the inclusion of educational materials. Table 1 presents a comprehensive comparison of all features as studied.

### Health Professional Involvement and Social Support

Among the 7 papers, 5 (71.43%) incorporated some form of connection between these apps and engagement with health care practitioners. For instance, in the study by Sutema et al [47], participants in the intervention group used app-based diet management software to input their daily dietary intake. Subsequently, a dietitian received these daily records and provided monthly dietary recommendations back to the patients. In the study by Takenga et al [45], therapy plans, instructions, and medical recommendations documented on patient portals were transmitted to users through the mobile app (Mobil Diab app). In the study by Poonprapai et al [50], a research pharmacist provided 3 to 5 infographics on diabetes education daily over 3 months. For the study by Esferjani et al [49], the intervention included access to a WhatsApp (Meta Platforms) group and training sessions conducted by health education and internal medicine experts. Lastly, Dugas et al [42] experimentally manipulated 4 different interventions, which included variations in the presence or absence of a provider during the app usage period.

Of the 7 studies, 3 (42.86%) of them incorporated built-in app features related to social support (ie, peer communication as described in the studies by Dugas et al [42] and Esferjani et al [49] or family support as in the study by Poonprapai et al [50]. Similar to the health provider involvement, out of these papers, only the study by Dugas et al [42] specifically varied conditions by including or excluding social support alongside the app.

### Attrition Rates and App Usage

Most studies did not report attrition rates. Of the included studies, only 3 studies [42,46,47] reported some information on app usage. As attrition can pose questions to the long-term effects of these mHealth app interventions, we reported here the information available. In the study by Dugas et al [42], older adults used the app for an average of 65.09 days over 13 weeks (SD 25.38 days). Or and Tao [46] reported that during the first month, older adults used their app for an average of 23 of 30 days, which decreased to 17 of 30 days by the third month. They found that older adults in both their app and control groups generally conducted self-measured blood pressure daily and self-measured BG 3 to 5 times per week, with no significant differences between the 2 groups. Ninety-one percent of older adults in their intervention group consistently used the self-monitoring system over the 3-month study period. System usage was robust in the initial month but declined in the subsequent months for both patient groups. During the first month, 28 of 30 (93%) patients uploaded measurements at least 3 days per week. This percentage decreased over time to 67% in the second month and to 73% in the third month. Lastly, Sutema et al [47] reported that after their trial ended, over 89% of patients in the intervention group continued to measure their BG level 2 to 3 days each week, which speaks to the level of engagement and usage of their app outside the scope of their RCT.

## Discussion

### Principal Findings

#### Overview

This meta-analysis indicated that interventions using mHealth apps through RCTs effectively enhanced diabetes-related outcomes among older adults, spanning various outcome measures. Moreover, the estimated effect sizes aligned with those observed in meta-analyses involving younger adults, a noteworthy finding in itself. There is often skepticism regarding the efficacy of mHealth interventions for older adults, presuming that these technologies might not be as beneficial for individuals less accustomed to them. However, our findings suggested that, at the very least, the examined apps demonstrated effectiveness comparable to those tested across a younger age range [10,24-29]. If anything, apps designed specifically for older adults might yield even greater effect sizes and more successful technology implementations to support their management of chronic conditions. Such advantages might be enhanced if mHealth apps are specifically designed with and tailored for older adults, rather than adopting a one-size-fits-all approach. A personalized and adaptive learning strategy can be investigated, as its effectiveness has been evidenced in other domains and contexts (eg, in the study by Alrawashdeh et al [53], reading literacy among young students).

In an era where technology integration in health care is increasingly prominent, particularly amid the shift toward remote care and digital health solutions, our research holds significant implications that transcend the scope of this study. This meta-analysis carries relevance not only for health care providers but also for researchers, policy makers, and stakeholders on designing and developing mHealth apps for older adults.

#### For Health Care Providers

Our findings underscored the potential of mHealth apps to enhance health outcomes among older adults across diverse health care settings. Providers seeking effective ways to leverage technology for improved patient care and health monitoring will find valuable insights within our study. We found that studies including some connection with health care providers [42,47,49,50] reported a larger effectiveness in reducing HbA_1c_ levels.

#### For Policy Makers

We emphasized the need to consider mHealth app interventions as a viable strategy to address health care challenges among older populations, particularly in regions with varying health care resources. By examining the varying outcomes among mHealth interventions across economically diverse regions and health care settings, this study underscored the importance of understanding the contexts in which these interventions are implemented. Although these findings were influenced by the quality of available evidence and the scarce number of RCTs in the area, they suggested practical implications for effective mHealth design to support health management across different health care environments. For example, future interventions should consider the usability and appropriateness of technology for older adults, ensuring compatibility with existing health care infrastructures.

#### For Researchers

Beyond the specific applications in health care for older adults, our work extends to the broader context of mHealth’s effectiveness and adaptability in various health environments. It serves as a foundational contribution, inviting further exploration of personalized technology’s impact on health management and beyond. For example, the meta-analysis by Robert et al [30] on the effectiveness of eHealth nutritional interventions found an overall positive improvement in FBG, body fat, triglyceride levels, and calorie intake for middle-aged and older adults. Our meta-analysis contributes to this literature by expanding the founded benefits to investigate apps designed for BG management. As our meta-analysis included only RCTs, we believe that our estimated effect-size should reflect the diverse components of mHealth apps beyond their educational content. Given that many of these educational components are also present in RCT control groups, the differential impact of these apps likely resides in other components absent from such controls. Researchers could further continue to explore and attempt to disentangle the educational benefits of these interventions from features related to self-management. We provided a summary of the components present in each of these app interventions to guide that exploration.

Moreover, dynamic and personalized educational materials should be explored as a way to continuously engage users, mitigate app abandonment, and augment the benefits observed by Robert et al [30] and the studies in our meta-analysis. The support components of a technology that can best support health goals are dependent on the patient’s journey [54], but the stage of diagnosis was not reported.

Some interventions did not demonstrate benefits, particularly the more dated RCTs in our sample, but the findings indicate that the use of mHealth may yield significant improvements compared to traditional health care methods, especially in blended care approaches. We positioned our study as foundational research that illuminated effective mHealth app intervention design for older adults and will serve as a catalyst for broader investigations into personalized technology’s impact on health care in our increasingly digital age.

### Strengths and Limitations

Our review had several strengths. It provided evidence regarding the effects of mobile app interventions developed for diabetes on patient outcomes tested with older adults. To our knowledge, this is the first to estimate the effectiveness of RCT interventions, answering if mHealth apps improve older adults’ diabetes management. Our review provides an evidence-based review of the features of such interventions and their associations with improvements in glycemic control and medication adherence.

Our study had limitations. This analysis drew upon RCT interventions conducted directly with older adults who adopted an mHealth app to improve outcome measures related to diabetes, investigating various populations across different diabetes types (1 and 2), education levels, and socioeconomic statuses in diverse settings. The RCTs included in this study exhibited moderate to high methodological quality despite potential limitations stemming from their small sample sizes, which might have influenced the overall representation of mHealth intervention progress. We attempted to circumvent this limitation by ordering our identified studies based on their publication chronology to provide readers with a way to identify potential progress as technology advances and sample sizes increase. As depicted in Figure 3, it is compelling that RCT interventions advanced over time, as shown by improvement in the assessment of biases across various dimensions. This suggests a promising trend toward the adoption of more rigorous RCT methodologies within the field.

Another potential limitation is the introduction of language bias, as we only screened papers published in English, ensuring that all screeners and coauthors could read the paper. While this is a common practice in meta-analyses, and it is likely that researchers publishing RCT results on digital technologies predominantly do so in English-language journals, we acknowledge the need for caution when interpreting conclusions and excluding studies published in other languages. Our sample also includes a diverse representation of countries, including China, the Democratic Republic of Congo, Hong Kong, Indonesia, Iran, Thailand, and the United States (see also [55]).

Specifically, this review highlighted the characteristics and outcomes of mHealth interventions for patients with diabetes. We did not explore interventions for other chronic illnesses such as hypertension, cholesterol, chronic obstructive pulmonary disease, arthritis, or liver diseases. In future studies, we will expand our approach to different chronic conditions and explore similarities and condition-specific features. Furthermore, terms such as telemonitoring and telemedicine, sometimes falling under the broader umbrella of mHealth, were not used in the literature search, and citation network analysis tools could be used in the future to enhance comprehensiveness.

Studies were heterogeneous in intervention duration, technology exposure, and outcome variable measures, making it difficult or sometimes infeasible to compare quantitatively across studies and generalize based on the length of the interventions. We circumvented this limitation by providing information on intervention duration length so a qualitative comparison could be inferred by readers. Our analyses were limited to the final effect based on these reported durations, and thus, future studies should take into account the minimum technology exposure time needed to observe some significant improvement as more studies and evidence accumulate in the literature. Our systematic review covered a broad range of databases but yielded no RCTs with a duration exceeding 9 months (see [50], included in our meta-analysis), and other meta-analyses on mHealth apps with younger adults also did not identify a study that exceeded beyond 1 year of usage. We recognize that RCTs are costly and challenging to conduct over long periods. However, a clear and actionable direction is the need for longitudinal studies on mHealth app usage. Future research should incorporate longitudinal points of contact with participants to assess whether the effects are sustained over time, explore mHealth abandonment, and investigate other behaviors that are more suited to the chronic nature of these health conditions.

Our search was not updated to include reviews published after December 2022, and we did not contact the authors of the identified papers to confirm if the published information was correct or to obtain more details about the interventions published. Although that would allow us to better identify characteristics, such as the unknown information reported in Table 1, we defended our choice in this case based on the idea that the information should be publicly available on published RCT papers, which are at the core of the replication principle in science.

### Conclusion

In conclusion, this meta-analysis provided compelling evidence that mHealth interventions using mobile apps significantly enhanced diabetes-related outcomes among older adults. Our findings not only underscored the effectiveness of these interventions across various outcome measures (ie, reduction in HbA_1c_ levels, Hedges g –0.40; increased medication adherence, g 1.06), but also challenged the skepticism surrounding their applicability to older populations. The observed effect sizes were comparable to those found in studies involving younger adults, suggesting promising potential for mHealth technologies in managing chronic conditions among older demographics. As health care increasingly adopts digital solutions, our research highlighted the significant role mHealth apps can play in enhancing patient care and monitoring, particularly in clinical practice. It also broadened the scope for researchers, urging further exploration into personalized technology for managing health, especially for older adults. Policy makers should consider mHealth apps as a strategy to address health care challenges, particularly in regions with varying health care resources. By examining outcomes across countries and economic contexts, our study highlighted the importance of tailoring interventions to be user-friendly and compatible with existing health care infrastructures. In summary, this meta-analysis serves as foundational research that not only validated the efficacy of mHealth apps in diabetes management for older adults but also stimulated ongoing dialogue and innovation in the field of digital health. As technology continues to evolve, so too will the opportunities to optimize health care delivery and improve outcomes for older adults through tailored and evidence-based interventions.

## Appendix

Multimedia Appendix 1 PRISMA (Preferred Reporting Items for Systematic Reviews and Meta-Analyses) 2020 checklist.

Multimedia Appendix 2 Query strings and general search strategy.

Multimedia Appendix 3 Publication bias and sensitivity analysis.

## Abbreviations

BG: blood glucose
FBG: fasting blood glucose
HbA1c: glycated hemoglobin
mHealth: mobile health
PRISMA: Preferred Reporting Items for Systematic Reviews and Meta-Analyses
RCT: randomized controlled trial
SPIDER: Sample, Phenomenon of Interest, Design, and Research

## Data Availability

The datasets generated or analyzed during this study are available from the corresponding author upon reasonable request.
