# Supplementary material for: Evaluating Effectiveness of mHealth Apps for Older Adults With Diabetes: Meta-Analysis of Randomized Controlled Trials
Source: J Med Internet Res. 2025 Jun 17;27:e65855. doi: 10.2196/65855 (PMC12214694; doi:10.2196/65855)
Supplement: Multimedia Appendix 1 [file jmir_v27i1e65855_app1.docx]

| **Section and Topic** | **Item #** | **Checklist item** | **Location where item is reported** |
| --- | --- | --- | --- |
| **TITLE** | | |  |
| Title | 1 | Identify the report as a systematic review. | Title |
| **ABSTRACT** | | |  |
| Abstract | 2 | See the PRISMA 2020 for Abstracts checklist. | See abstract checklist below |
| **INTRODUCTION** | | |  |
| Rationale | 3 | Describe the rationale for the review in the context of existing knowledge. | Significance section, pp. 3-4 |
| Objectives | 4 | Provide an explicit statement of the objective(s) or question(s) the review addresses. | Research Objective section, p. 4 |
| **METHODS** | | |  |
| Eligibility criteria | 5 | Specify the inclusion and exclusion criteria for the review and how studies were grouped for the syntheses. | Inclusion and Exclusion criteria section, p. 5 |
| Information sources | 6 | Specify all databases, registers, websites, organisations, reference lists and other sources searched or consulted to identify studies. Specify the date when each source was last searched or consulted. | Design, Data Sources, and Literature Search Strategy section, pp. 4-5 |
| Search strategy | 7 | Present the full search strategies for all databases, registers and websites, including any filters and limits used. | Design, Data Sources, and Literature Search Strategy section, pp. 4-5   Appendix A |
| Selection process | 8 | Specify the methods used to decide whether a study met the inclusion criteria of the review, including how many reviewers screened each record and each report retrieved, whether they worked independently, and if applicable, details of automation tools used in the process. | Study screening and selection section, pp. 5-6 |
| Data collection process | 9 | Specify the methods used to collect data from reports, including how many reviewers collected data from each report, whether they worked independently, any processes for obtaining or confirming data from study investigators, and if applicable, details of automation tools used in the process. | Study screening and selection section, pp. 5-6 |
| Data items | 10a | List and define all outcomes for which data were sought. Specify whether all results that were compatible with each outcome domain in each study were sought (e.g. for all measures, time points, analyses), and if not, the methods used to decide which results to collect. | Outcome measures section and Data extraction, synthesis, and analysis section, pp. 6-7 Also: see App feature analysis, p. 9 |
|  | 10b | List and define all other variables for which data were sought (e.g. participant and intervention characteristics, funding sources). Describe any assumptions made about any missing or unclear information. | Outcome measures section and Data extraction, synthesis, and analysis section, pp. 6-7  Also: see Table 1 |
| Study risk of bias assessment | 11 | Specify the methods used to assess risk of bias in the included studies, including details of the tool(s) used, how many reviewers assessed each study and whether they worked independently, and if applicable, details of automation tools used in the process. | Critical Appraisal (Assessment of Quality of Evidence and Risk of Biases) section, p. 7-9 Also: see Appendix B |
| Effect measures | 12 | Specify for each outcome the effect measure(s) (e.g. risk ratio, mean difference) used in the synthesis or presentation of results. | Outcome measures section and Data extraction, synthesis, and analysis section, pp. 6-7  Also see: Results: “Glycated Hemoglobin (HbA1c)”, “Fasting Blood Sugar (FBS)”,” Medication Adherence”, and “Other diabetes-related outcome measures” sections, pp. 13-16 |
| Synthesis methods | 13a | Describe the processes used to decide which studies were eligible for each synthesis (e.g. tabulating the study intervention characteristics and comparing against the planned groups for each synthesis (item #5)). | Data extraction, synthesis, and analysis section, pp. 6-7  Also see: Results: “Glycated Hemoglobin (HbA1c)”, “Fasting Blood Sugar (FBS)”,” Medication Adherence”, and “Other diabetes-related outcome measures” sections, pp. 13-16 |
|  | 13b | Describe any methods required to prepare the data for presentation or synthesis, such as handling of missing summary statistics, or data conversions. | Data extraction, synthesis, and analysis section, pp. 6-7 |
|  | 13c | Describe any methods used to tabulate or visually display results of individual studies and syntheses. | Data extraction, synthesis, and analysis section, pp. 6-7  Also see: Tables 1 and 2 |
|  | 13d | Describe any methods used to synthesize results and provide a rationale for the choice(s). If meta-analysis was performed, describe the model(s), method(s) to identify the presence and extent of statistical heterogeneity, and software package(s) used. | Data extraction, synthesis, and analysis section, pp. 6-7 |
|  | 13e | Describe any methods used to explore possible causes of heterogeneity among study results (e.g. subgroup analysis, meta-regression). | Data extraction, synthesis, and analysis section, pp. 6-7  Appendix B  Also see: Results: “Glycated Hemoglobin (HbA1c)”, “Fasting Blood Sugar (FBS)”,” and Medication Adherence”, pp. 13-16 |
|  | 13f | Describe any sensitivity analyses conducted to assess robustness of the synthesized results. | Appendix B |
| Reporting bias assessment | 14 | Describe any methods used to assess risk of bias due to missing results in a synthesis (arising from reporting biases). | Critical Appraisal (Assessment of Quality of Evidence and Risk of Biases) section, p. 7-9 Also: see Appendix B |
| Certainty assessment | 15 | Describe any methods used to assess certainty (or confidence) in the body of evidence for an outcome. | Critical Appraisal (Assessment of Quality of Evidence and Risk of Biases) section, p. 7-9 Also: see Appendix B |
| **RESULTS** | | |  |
| Study selection | 16a | Describe the results of the search and selection process, from the number of records identified in the search to the number of studies included in the review, ideally using a flow diagram. | Study selection, p.10  Also see: Figure 1, p. 6 |
|  | 16b | Cite studies that might appear to meet the inclusion criteria, but which were excluded, and explain why they were excluded. | Study selection, p.10  Also see: Figure 1, p. 6 |
| Study characteristics | 17 | Cite each included study and present its characteristics. | Tables 1 and 2  Figures 4, 5, 6 Results: “Glycated Hemoglobin (HbA1c)”, “Fasting Blood Sugar (FBS)”,” Medication Adherence”, and “Other diabetes-related outcome measures” sections, pp. 13-16 |
| Risk of bias in studies | 18 | Present assessments of risk of bias for each included study. | Figures 2 and 3  Also: information available and integrated on Figures 4, 5, and 6. |
| Results of individual studies | 19 | For all outcomes, present, for each study: (a) summary statistics for each group (where appropriate) and (b) an effect estimate and its precision (e.g. confidence/credible interval), ideally using structured tables or plots. | Tables 1 and 2  Figures 4, 5, 6 Results: “Glycated Hemoglobin (HbA1c)”, “Fasting Blood Sugar (FBS)”,” Medication Adherence”, and “Other diabetes-related outcome measures” sections, pp. 13-16 |
| Results of syntheses | 20a | For each synthesis, briefly summarise the characteristics and risk of bias among contributing studies. | Critical Appraisal (Assessment of Quality of Evidence and Risk of Biases) section, p. 7-9  Figures 4, 5, and 6 |
|  | 20b | Present results of all statistical syntheses conducted. If meta-analysis was done, present for each the summary estimate and its precision (e.g. confidence/credible interval) and measures of statistical heterogeneity. If comparing groups, describe the direction of the effect. | Figures 4, 5, and 6  Results: “Glycated Hemoglobin (HbA1c)”, “Fasting Blood Sugar (FBS)”,” Medication Adherence”, and “Other diabetes-related outcome measures” sections, pp. 13-16 |
|  | 20c | Present results of all investigations of possible causes of heterogeneity among study results. | Results: “Glycated Hemoglobin (HbA1c)”, “Fasting Blood Sugar (FBS)”,” Medication Adherence”, and “Other diabetes-related outcome measures” sections, pp. 13-16  Also see: Appendix B |
|  | 20d | Present results of all sensitivity analyses conducted to assess the robustness of the synthesized results. | Appendix B |
| Reporting biases | 21 | Present assessments of risk of bias due to missing results (arising from reporting biases) for each synthesis assessed. | Critical Appraisal (Assessment of Quality of Evidence and Risk of Biases) section, p. 7-9 Also Appendix B |
| Certainty of evidence | 22 | Present assessments of certainty (or confidence) in the body of evidence for each outcome assessed. | Critical Appraisal (Assessment of Quality of Evidence and Risk of Biases) section, p. 7-9  Also: Appendix B |
| **DISCUSSION** | | |  |
| Discussion | 23a | Provide a general interpretation of the results in the context of other evidence. | Principal findings, p. 18-19 |
|  | 23b | Discuss any limitations of the evidence included in the review. | Strengths and limitations, p. 19-20 |
|  | 23c | Discuss any limitations of the review processes used. | Strengths and limitations, p. 19-20 |
|  | 23d | Discuss implications of the results for practice, policy, and future research. | Principal findings, p. 18-19 Also: Conclusion |
| **OTHER INFORMATION** | | |  |
| Registration and protocol | 24a | Provide registration information for the review, including register name and registration number, or state that the review was not registered. | Open Science Framework (OSF) Registries:  <http://doi.org/10.17605/OSF.IO/AWVCX>.  Methods: Design, Data Sources, and Literature Search |
|  | 24b | Indicate where the review protocol can be accessed, or state that a protocol was not prepared. | Open Science Framework (OSF) Registries:  <http://doi.org/10.17605/OSF.IO/AWVCX>.  Methods: Design, Data Sources, and Literature Search |
|  | 24c | Describe and explain any amendments to information provided at registration or in the protocol. | N/A |
| Support | 25 | Describe sources of financial or non-financial support for the review, and the role of the funders or sponsors in the review. | Acknowledgments section |
| Competing interests | 26 | Declare any competing interests of review authors. | Conflicts of Interest section: None declared. |
| Availability of data, code and other materials | 27 | Report which of the following are publicly available and where they can be found: template data collection forms; data extracted from included studies; data used for all analyses; analytic code; any other materials used in the review. | Data Availability section |

| **Section and Topic** | **Item #** | **Checklist item** | **Reported (Yes/No)** |
| --- | --- | --- | --- |
| **TITLE** | | |  |
| Title | 1 | Identify the report as a systematic review. | Yes |
| **BACKGROUND** | | |  |
| Objectives | 2 | Provide an explicit statement of the main objective(s) or question(s) the review addresses. | Yes |
| **METHODS** | | |  |
| Eligibility criteria | 3 | Specify the inclusion and exclusion criteria for the review. | Yes |
| Information sources | 4 | Specify the information sources (e.g. databases, registers) used to identify studies and the date when each was last searched. | No: Reported in the paper |
| Risk of bias | 5 | Specify the methods used to assess risk of bias in the included studies. | No: Reported in the paper |
| Synthesis of results | 6 | Specify the methods used to present and synthesize results. | Yes |
| **RESULTS** | | |  |
| Included studies | 7 | Give the total number of included studies and participants and summarize relevant characteristics of studies. | Yes |
| Synthesis of results | 8 | Present results for main outcomes, preferably indicating the number of included studies and participants for each. If meta-analysis was done, report the summary estimate and confidence/credible interval. If comparing groups, indicate the direction of the effect (i.e. which group is favored). | Yes |
| **DISCUSSION** | | |  |
| Limitations of evidence | 9 | Provide a brief summary of the limitations of the evidence included in the review (e.g. study risk of bias, inconsistency and imprecision). | No: Reported in the paper |
| Interpretation | 10 | Provide a general interpretation of the results and important implications. | Yes |
| **OTHER** | | |  |
| Funding | 11 | Specify the primary source of funding for the review. | No: Reported in the paper |
| Registration | 12 | Provide the register name and registration number. | No: Reported in the paper |

*From:*  Page MJ, McKenzie JE, Bossuyt PM, Boutron I, Hoffmann TC, Mulrow CD, et al. The PRISMA 2020 statement: an updated guideline for reporting systematic reviews. BMJ 2021;372:n71. doi: 10.1136/bmj.n71. This work is licensed under CC BY 4.0. To view a copy of this license, visit <https://creativecommons.org/licenses/by/4.0/>
