# Supplementary material for: Evaluating Effectiveness of mHealth Apps for Older Adults With Diabetes: Meta-Analysis of Randomized Controlled Trials
Source: J Med Internet Res. 2025 Jun 17;27:e65855. doi: 10.2196/65855 (PMC12214694; doi:10.2196/65855)
Supplement: Multimedia Appendix 2 [file jmir_v27i1e65855_app2.docx]

1. Mobile health applications (combine using OR)

• App

• Cell Phone

• Digital technolog*

• Mobile

• mHealth app

• Mobile Application

• Smartphone

• Phone

AND

2. Age-related terms (combine using OR)

• Age

• Aged

• Aging

• Elderly

• Geriatric*

• Older adult*

• Seniors

AND

3. Health condition-related (Diabetes) terms (combine using OR)

• Diabet*

• Gluco*

• Glycemi*

• Insulin

• Mellitus

• Sugar

• A1C

• Hemoglobin

For a complete list of query strings for each data, see: <https://doi.org/10.17605/OSF.IO/AWVCX>
