# Supplementary material for: Evaluating Effectiveness of mHealth Apps for Older Adults With Diabetes: Meta-Analysis of Randomized Controlled Trials
Source: J Med Internet Res. 2025 Jun 17;27:e65855. doi: 10.2196/65855 (PMC12214694; doi:10.2196/65855)
Supplement: Multimedia Appendix 3 [file jmir_v27i1e65855_app3.docx]

The funnel plot (Figure S1) provides a visual representation of the relationship between the effect sizes of individual studies and their precision as measured by standard error. We present this analysis exclusively for the HbA1c measure, as it is the outcome reported by the majority of the studies (6 papers with 9 RCT interventions).


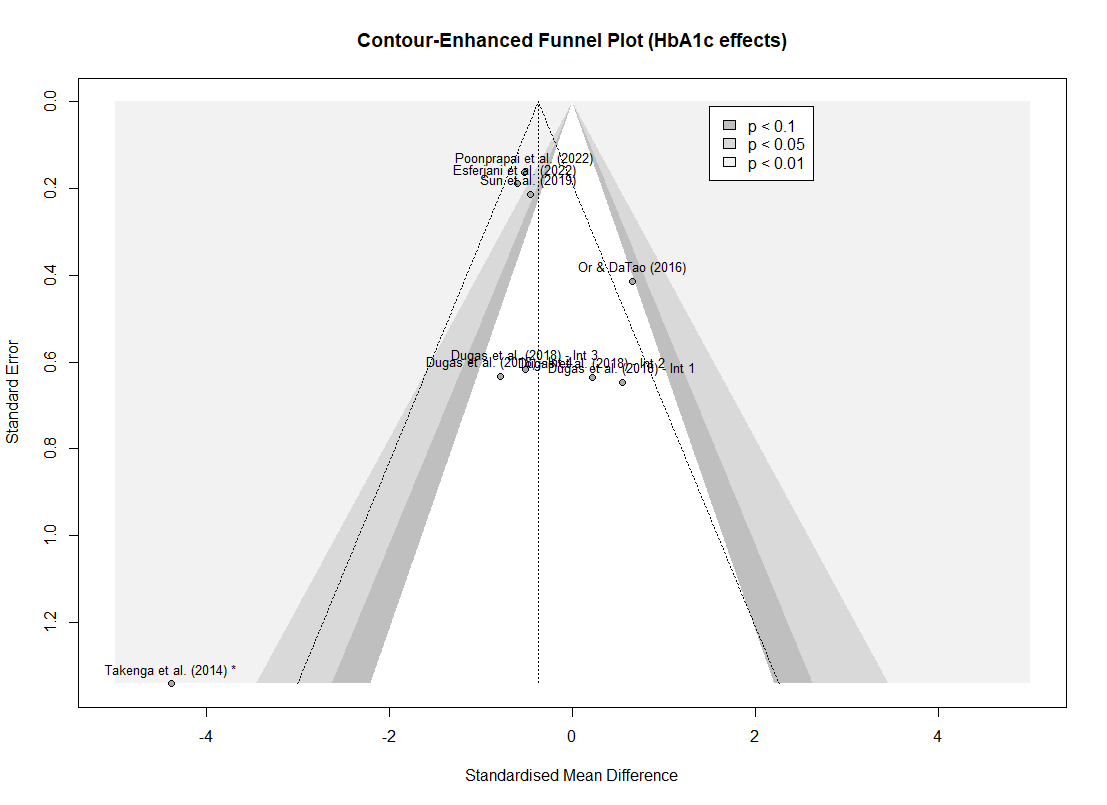


**Figure S1.** Funnel plot of standard error by standardized mean differences on the effects on HbA1c. HbA1c measures provided for 9 studies

While our plot reveals a relatively asymmetrical distribution of studies around the average: 3 to the right of the line, 6 to the left of the line, the Begg and Mazumdar correlation test [53] (p = .677) and the Egger’s test of the intercept [54] were not significant (t = 0.140, df = 7, p-value = .891). Hence, the visual asymmetry conveyed is probably not caused by file- drawing (unpublished RCT studies not showing benefits of mHealth applications), but more likely due to the small number of study effects. For the sake of completeness, we also conducted a Duval and Tweedie’s trim and fill test [55] to account for the potential impact of publication bias on meta-analytic results by estimating and adjusting for missing studies. We estimate the effect size that would have been observed if all studies, regardless of their results, had been published. The Duval and Tweedie’s test [55] under the random effects model, yields a point estimate for the combined studies of -0.376 (95% confidence interval: -0.711 to -0.04), using the trim and fill function [56], trimfill(), *metafor* package), these values remain unchanged. Hence, we found no evidence that publication biases could impact the interpretation of the observed intervention effect sizes estimated. The spread of points in the funnel plot also help to indicate the heterogeneity among the included studies. In particularly, [43] shows an effect size beyond 4 units of the standardized mean difference, a variability of effect size that is perhaps beyond what would be expected by chance alone. Hence, we suggest caution on the interpretation of findings for this study in particularly. While cultural differences, small sample of participants in this study, and different technologies might help to explain differences in effect sizes between mHealth interventions and usual care control conditions, our study helps to estimate the most probable likelihood that mHealth applications have an effectiveness beyond the usual care.
